# Supplementary material for: Safe infant feeding in healthcare facilities: Assessment of infection prevention and control conditions and behaviors in India, Malawi, and Tanzania
Source: PLOS Glob Public Health. 2023 Jun 8;3(6):e0001843. doi: 10.1371/journal.pgph.0001843 (PMC10249877; doi:10.1371/journal.pgph.0001843)
Supplement: S1 Table — (DOCX) [file pgph.0001843.s001.docx]

**Supplemental Material:**

**Safe infant feeding in healthcare facilities: Assessment of infection prevention and control conditions and behaviors in India, Malawi, and Tanzania**

Bethany A. Caruso^1^, Uriel Paniagua^2^, Irving Hoffman^3^, Karim Manji^4^, Friday Saidi^5^, Christopher R. Sudfeld^6^, Sunil S Vernekar^7^, Mohamed Bakari^4^, Christopher P. Duggan^8^, George C. Kibogoyo^4^, Rodrick Kisenge^4^, Sarah Somji^4^, Eddah Kafansiyanji^5^, Tisungane Mvalo^5,10,^, Naomie Nyirenda^5^, Melda Phiri^5^, Roopa Bellad^7^, Sangappa Dhaded^7^, Chaya K A^11^, Bhavana Koppad^7^, Shilpa Nabapure^12^, Saumya Nanda^13^, Bipsa Singh^13^, S Yogeshkumar^7^, Katelyn Fleming^14^, Krysten North^15^, Danielle E Tuller^14^, Katherine EA Semrau^14,16^, Linda Vesel^14^, Melissa F Young^1^, and the LIFE Study Team

1. Hubert Department of Global Health, Emory University School of Public Health, Atlanta, Georgia, USA. 2. Department of Epidemiology, Emory University School of Public Health, Atlanta, Georgia, USA. 3. Institute for Global Health and Infectious Diseases, University of North Carolina at Chapel Hill School of Medicine, Chapel Hill, North Carolina, USA. 4. Department of Pediatrics and Child Health, Muhimbili University of Health and Allied Sciences, Dar es Salaam, Tanzania. 5. University of North Carolina Project Malawi, Lilongwe, Malawi. 6. Department of Global Health and Population, Harvard T.H. Chan School of Public Health, Boston, Massachusetts, USA. 7. Jawaharlal Nehru Medical College, KLE Academy of Higher Education and Research (Deemed-to-be-University), Belgaum, Karnataka, India. 8. Center for Nutrition, Boston Children's Hospital, Boston, Massachusetts, USA. 10. Department of Pediatrics, University of North Carolina at Chapel Hill School of Medicine, Chapel Hill, North Carolina, USA. 11. Bapuji Child Health Institute & Research Centre, Davangere, Karnataka, India. 12. SS Institute of Medical Sciences & Research Centre, Davangere, Karnataka, India. 13. Shri Jagannath Medical College and Hospital, Puri, Odisha, India. 14. Ariadne Labs, Harvard T.H. Chan School of Public Health / Brigham and Women's Hospital, Boston, Massachusetts, USA. 15. Brigham and Women’s Hospital and Harvard Medical School, Boston, Massachusetts, USA. 16. Harvard Medical School, Boston, MA, USA.

**Abstract**

Infants need to receive care in environments that limit their exposure to pathogens. Inadequate water, sanitation, and hygiene (WASH) environments and suboptimal infection prevention and control practices in healthcare settings contribute to the burden of healthcare-associated infections, which are particularly high in low-income settings. Specific research is needed to understand infant feeding preparation in healthcare settings, a task involving multiple behaviors that can introduce pathogens and negatively impact health. To understand feeding preparation practices and potential risks, and to inform strategies for improvement, we assessed facility WASH environments and observed infant feeding preparation practices across 12 facilities in India, Malawi, and Tanzania serving newborn infants. Research was embedded within the Low Birthweight Infant Feeding Exploration (LIFE) observational cohort study, which documented feeding practices and growth patterns to inform feeding interventions. We assessed WASH-related environments and feeding policies of all 12 facilities involved in the LIFE study. Additionally, we used a guidance-informed tool to carry out 27 feeding preparation observations across 9 facilities, enabling assessment of 270 total behaviors. All facilities had ‘improved’ water and sanitation services. Only 50% had written procedures for preparing expressed breastmilk; 50% had written procedures for cleaning, drying, and storage of infant feeding implements; and 33% had written procedures for preparing infant formula. Among 270 behaviors assessed across the 27 feeding preparation observations, 46 (17.0%) practices were carried out suboptimally, including preparers not handwashing prior to preparation, and cleaning, drying, and storing of feeding implements in ways that do not effectively prevent contamination. While further research is needed to improve assessment tools and to identify specific microbial risks of the suboptimal behaviors identified, the evidence generated is sufficient to justify investment in developing guidance and programing to strengthen infant feeding preparation practices to ensure optimal newborn health.

**Safe infant feeding in healthcare facilities: Assessment of infection prevention and control conditions and behaviors in India, Malawi, and Tanzania: Supplemental Materials**

**S1 Table.** Direct Observations of WASH Practices across and within Facilities.^1^

|  | | **Facility (Observations)** | | | | | | | | | | | | | | | | | |  | | | |
| --- | --- | --- | --- | --- | --- | --- | --- | --- | --- | --- | --- | --- | --- | --- | --- | --- | --- | --- | --- | --- | --- | --- | --- |
|  | | **A (N=4)** | | **B (N=1)** | | **C (N=1)** | | **D (N=5)** | | **E (N=2)** | | **F (N=6)** | | **G (N=1)** | | **H (N=4)** | | **I (N=3)** | | **Overall (N=27)** | | | |
| **a. Preparation room water source** |  | |  | |  | |  | |  | |  | |  | |  | |  | |  | | | |  |
| Water stored in room from outside source | 4 (100%) | | - | | 1 (100%) | | - | | - | | - | | - | | - | | 3 (100%) | | 8 (29.6%) | | | |  |
| Functional piped water into room* | - | | 1 (100%) | | - | | 5 (100%) | | 2 (100%) | | 6 (100%) | | 1 (100%) | | 4 (100%) | | - | | 19 (70.4%) | | | |  |
| **b. How is water treated?^1^** |  | |  | |  | |  | |  | |  | |  | |  | |  | |  | | | |  |
| Boiled prior to use* | 4 (100%) | | 1 (100%) | | 1 (100%) | | - | | 1 (50.0%) | | 6 (100%) | | - | | 3 (75.0%) | | - | | 16 (59.3%) | | | |  |
| Filtered prior to use* | - | | - | | - | | 5 (100%) | | - | | - | | - | | - | | - | | 5 (18.5%) | | | |  |
| N/A – Bottled/sachet water* | - | | - | | 1 (100%) | | - | | 1 (50.0%) | | - | | - | | - | | 3 (100%) | | 5 (18.5%) | | | |  |
| Treated with chlorine/bleach prior to use* | - | | - | | - | | - | | - | | - | | 1 (100%) | | - | | - | | 1 (3.7%) | | | |  |
| Missing | - | | - | | - | | - | | - | | - | | - | | 1 (25.0%) | | - | | 1 (3.7%) | | | |  |
| **c. Does the person preparing the feed wash their hands before starting any feed preparation activities?** | | | | | | | | | | | | | | | | | | | | |  |  |  |
| Yes, with soap and water* | 1 (25.0%) | | 1 (100%) | | 1 (100%) | | 5 (100%) | | 1 (50.0%) | | 6 (100%) | | 1 (100%) | | 4 (100%) | | 3 (100%) | | 23 (85.2%) | | | |  |
| Yes, with only water | 1 (25.0%) | | - | | - | | - | | - | | - | | - | | - | | - | | 1 (3.7%) | | | |  |
| No | 2 (50.0%) | | - | | - | | - | | - | | - | | - | | - | | - | | 2 (7.4%) | | | |  |
| Not observed | - | | - | | - | | - | | 1 (50.0%) | | - | | - | | - | | - | | 1 (3.7%) | | | |  |
| **d. Were any of the feeding supplies cleaned immediately before use?** | | | | | | | | | | | | | | | | | | | | |  |  |  |
| Yes, washed with WATER AND SOAP by hand immediately before use* | 3 (75.0%) | | - | | 1 (100%) | | 1 (20.0%) | | 1 (50.0%) | | 6 (100%) | | 1 (100%) | | 4 (100%) | | 3 (100%) | | 20 (74.1%) | | | |  |
| Yes, washed with WATER ONLY by hand immediately before use | - | | - | | - | | 4 (80.0%) | | 1 (50.0%) | | - | | - | | - | | - | | 5 (18.5%) | | | |  |
| No, not washed immediately before use. | 1 (25.0%) | | 1 (100%) | | - | | - | | - | | - | | - | | - | | - | | 2 (7.4%) | | | |  |
| **e. What is used to wash feeding supplies prior to use?** | | | | | | | | | | | | | | | | | | | | |  |  |  |
| New/not previously used sponge/brush* | 2 (50.0%) | | - | | - | | - | | - | | 4 (66.7%) | | - | | - | | - | | 6 (22.2%) | | | |  |
| Previously used sponge/brush that has been sterilized* | - | | - | | - | | 4 (80.0%) | | - | | 2 (33.3%) | | 1 (100%) | | 4 (100%) | | 2 (66.7%) | | 13 (48.1%) | | | |  |
| Previously used sponge/brush, NOT sterilized | - | | - | | - | | 1 (20.0%) | | - | | - | | - | | - | | - | | 1 (3.7%) | | | |  |
| Other, unspecified | - | | - | | 1 (100%) | | - | | - | | - | | - | | - | | - | | 1 (3.7%) | | | |  |
| Not observed | 1 (25.0%) | | - | | - | | - | | 2 (100%) | | - | | - | | - | | 1 (33.3%) | | 4 (14.8%) | | | |  |
| Feeding supplies not washed before use | 1 (25.0%) | | 1 (100%) | | - | | - | | - | | - | | - | | - | | - | | 2 (7.4%) | | | |  |
| **f. Were the feeding supplies dry before use?** |  | |  | |  | |  | |  | |  | |  | |  | |  | |  | | | |  |
| Yes, feeding supplies dried with cloth before use | - | | - | | - | | - | | - | | - | | - | | - | | 3 (100%) | | 3 (11.1%) | | | |  |
| Yes, feeding supplies dried with paper/disposable towel before use* | - | | - | | - | | 2 (40.0%) | | - | | - | | 1 (100%) | | - | | - | | 3 (11.1%) | | | |  |
| Yes, feeding supplies taken from rack where air dried before use* | - | | 1 (100%) | | - | | - | | 1 (50.0%) | | 6 (100%) | | - | | 4 (100%) | | - | | 12 (44.4%) | | | |  |
| Yes, feeding supplies dry but method not observed | - | | - | | - | | 3 (60.0%) | | 1 (50.0%) | | - | | - | | - | | - | | 4 (14.8%) | | | |  |
| No, feeding supplies not fully dry before use (some or all still wet) | 4 (100%) | | - | | 1 (100%) | | - | | - | | - | | - | | - | | - | | 5 (18.5%) | | | |  |
| **g. Are the feeding supplies cleaned after use?** |  | |  | |  | |  | |  | |  | |  | |  | |  | |  | | | |  |
| Yes, feeding supplies washed by hand with WATER AND SOAP after use* | 3 (75.0%) | | 1 (100%) | | 1 (100%) | | 5 (100%) | | 1 (50.0%) | | 6 (100%) | | 1 (100%) | | 4 (100%) | | 3 (100%) | | 25 (92.6%) | | | |  |
| Yes, feeding supplies washed by hand with WATER ONLY after use | 1 (25.0%) | | - | | - | | - | | - | | - | | - | | - | | - | | 1 (3.7%) | | | |  |
| Not observed | - | | - | | - | | - | | 1 (50.0%) | | - | | - | | - | | - | | 1 (3.7%) | | | |  |
| **h. Are feeding supplies dried before storage or next use?** | | | | | | | | | | | | | | | | | | | | | |  |  |
| Yes, feeding supplies dried as part of drying cycle in dishwasher machine prior to storage or next use* | - | | - | | - | | - | | - | | - | | 1 (100%) | | - | | 1 (33.3%) | | 2 (7.4%) | | | |  |
| Yes, feeding supplies air drying in rack for before storage or next use* | - | | 1 (100%) | | - | | 2 (40.0%) | | 1 (50.0%) | | 6 (100%) | | - | | 4 (100%) | | - | | 14 (51.9%) | | | |  |
| Yes, feeding supplies dried with paper/disposable towel before storage or next use* | - | | - | | - | | 3 (60.0%) | | - | | - | | - | | - | | - | | 3 (11.1%) | | | |  |
| Yes, feeding supplies dried with cloth before storage or next use | - | | - | | - | | - | | - | | - | | - | | - | | 2 (66.7%) | | 2 (7.4%) | | | |  |
| No, feeding supplies NOT dried prior to storage or next use | 4 (100%) | | - | | 1 (100%) | | - | | - | | - | | - | | - | | - | | 5 (18.5%) | | | |  |
| Not observed | - | | - | | - | | - | | 1 (50.0%) | | - | | - | | - | | - | | 1 (3.7%) | | | |  |
| **i. Are feeding supplies placed in sterile bags/pouches after cleaning to sustain cleanliness for next use?** | | | | | | | | | | | | | | | | | | | | | |  |  |
| Yes* | 4 (100%) | | - | | - | | 2 (40.0%) | | - | | 6 (100%) | | 1 (100%) | | 4 (100%) | | 3 (100%) | | 20 (74.1%) | | | |  |
| No | - | | 1 (100%) | | 1 (100%) | | 3 (60.0%) | | 1 (50.0%) | | - | | - | | - | | - | | 6 (22.2%) | | | |  |
| Not observed | - | | - | | - | | - | | 1 (50.0%) | | - | | - | | - | | - | | 1 (3.7%) | | | |  |
| **j. Where were feeding supplies stored when not in use?** | | | | | | | | | | | | | | | | | | | | | |  |  |
| Closed bucket* | 4 (100%) | | - | | - | | - | | - | | - | | - | | - | | - | | 4 (14.8%) | | | |  |
| On a tray covered with clean cloth* | - | | 1 (100%) | | - | | - | | - | | - | | - | | - | | - | | 1 (3.7%) | | | |  |
| Out in open | - | | - | | 1 (100%) | | 5 (100%) | | - | | - | | - | | - | | - | | 6 (22.2%) | | | |  |
| In cabinets* | - | | - | | - | | - | | 2 (100%) | | 6 (100%) | | 1 (100%) | | 1 (25.0%) | | - | | 10 (37.0%) | | | |  |
| Sterile container (Bucket with lid)* | - | | - | | - | | - | | - | | - | | - | | 3 (75.0%) | | - | | 3 (11.1%) | | | |  |
| Sterile steel box* | - | | - | | - | | - | | - | | - | | - | | - | | 3 (100%) | | 3 (11.1%) | | | |  |
| **Does the person preparing the feed put on gloves before starting any feed preparation activities?** | | | | | | | | | | | | | | | | | | | | | |  |  |
| Yes | 2 (50.0%) | | - | | - | | - | | 1 (50.0%) | | 4 (66.7%) | | 1 (100%) | | - | | 3 (100%) | | 11 (40.7%) | | | |  |
| No | 2 (50.0%) | | 1 (100%) | | 1 (100%) | | 5 (100%) | | 1 (50.0%) | | 2 (33.3%) | | - | | 3 (75.0%) | | - | | 15 (55.6%) | | | |  |
| Not observed | - | | - | | - | | - | | - | | - | | - | | 1 (25.0%) | | - | | 1 (3.7%) | | | |  |
| **What type of feed is being prepared?^2^** |  | |  | |  | |  | |  | |  | |  | |  | |  | |  | | | |  |
| Formula | 4 (100%) | | 1 (100%) | | 1 (100%) | | 3 (60.0%) | | 2 (100%) | | 5 (83.3%) | | - | | 4 (100%) | | 3 (100%) | | 23 (85.2%) | | | |  |
| Breastmilk | - | | - | | 1 (100%) | | 2 (40.0%) | | - | | 2 (33.3%) | | 1 (100%) | | - | | - | | 6 (22.2%) | | | |  |
| **Feed preparation location** |  | |  | |  | |  | |  | |  | |  | |  | |  | |  | | | |  |
| Patient room | 4 (100%) | | 1 (100%) | | 1 (100%) | | - | | - | | - | | - | | - | | - | | 6 (22.2%) | | | |  |
| Kitchen | - | | - | | - | | 5 (100%) | | - | | 4 (66.7%) | | - | | 4 (100%) | | - | | 13 (48.1%) | | | |  |
| Nurses station | - | | - | | - | | - | | 2 (100%) | | - | | - | | - | | - | | 2 (7.4%) | | | |  |
| Break room | - | | - | | - | | - | | - | | 2 (33.3%) | | - | | - | | 1 (33.3%) | | 3 (11.1%) | | | |  |
| Milk preparation area | - | | - | | - | | - | | - | | - | | - | | - | | 2 (66.7%) | | 2 (7.4%) | | | |  |
| **Person preparing feed^2^** |  | |  | |  | |  | |  | |  | |  | |  | |  | |  | | | |  |
| Nurse | 4 (100%) | | 1 (100%) | | 1 (100%) | | 5 (100%) | | 2 (100%) | | 6 (100%) | | 1 (100%) | | 4 (100%) | | 3 (100%) | | 27 (100%) | | | |  |
| Nurse assistant | - | | - | | - | | 1 (20.0%) | | - | | 6 (100%) | | 1 (100%) | | - | | - | | 8 (29.6%) | | | |  |

1. a-j correspond to guidance noted in Table 1 and columns in Figure 1.
2. Multiple responses possible.

* Optimal response according to guidance (for items a-j only).
