# Supplementary material for: Safe infant feeding in healthcare facilities: Assessment of infection prevention and control conditions and behaviors in India, Malawi, and Tanzania
Source: PLOS Glob Public Health. 2023 Jun 8;3(6):e0001843. doi: 10.1371/journal.pgph.0001843 (PMC10249877; doi:10.1371/journal.pgph.0001843)
Supplement: Attachment — Submitted filename: PLOS GPH LIFE IPC Response to Reviewers 1MAR23.docx [file pgph.0001843.s003.docx]

**Response to reviewers**

**Submission:** PGPH-D-22-01747

We thank the reviewers for their comments. The suggestions were valuable and the revisions made have strengthened this publication. We provide a point-by-point response to reviewers’ comments and a revised version of our manuscript.

| **Editorial Comments** | | |
| --- | --- | --- |
| **Comments / requested revisions** | **Authors’ response** | **Additions /edits to manuscript (as applicable)** |
| E1.  Thank you for submitting your manuscript to PLOS Global Public Health. After careful consideration, we feel that it has merit but does not fully meet PLOS Global Public Health’s publication criteria as it currently stands. Therefore, we invite you to submit a revised version of the manuscript that addresses the points raised during the review process. | E1.  We appreciate the opportunity to revise and have addressed all comments from the Editor and Reviewers below. | |
| E2a.  a. In the methods, please clarify if an existing tool was used or adapted to guide data collection for the facility needs assessment (e.g., WHO IPC Readiness Assessment tool). | E2.  a. In the intro section we, point out that there are tools to assess and guide improvements in WASH and IPC in HCFs (with citations to example), though there are no tools specifically for assessing IPC related to behaviors for infant feeding, and thus these are needed. The noted WHO IPC Readiness Assessment tool was not one referenced, but one we now cite. Like the other tools we found, the WHO tool is a valuable facility level tool, but does not focus on specific assessment of infant feeding behaviors.  In the methods section we already describe the items in our tool and how they were informed. Specifically, we note that some questions for our tool come directly from a WHO/UNICEF tool:  “Questions used to assess facility-level water and sanitation are among the ‘core questions’ used for global monitoring of water and sanitation in healthcare facilities by the WHO/UNICEF Joint Monitoring Programme for Water Supply, Sanitation and Hygiene for global monitoring.”  In the methods, we already describe the observation tool and how, because no similar tool already existed, it was adapted based on guidelines for infection prevention and control related to breast pump milk collection kits: “There is no standardized tool for assessing infant feed preparation in healthcare facilities. As such, we created feeding preparation observation prompts based on relevant guidance from the Joint Working Group of the Healthcare Infection Society & Infection Prevention Society on the decontamination of breast pump milk collection kits and related items at home and in hospital.(36)  Given that the guidance was created for and informed by practitioners in the United Kingdom, observation prompts were added or adapted to fit the context and research scope. For example, Guideline 1.2 emphasizes the importance of ‘good microbiological quality water’ where mothers express milk and for rinsing of feeding-related implements. Microbial water quality assessment was not possible, thus questions about water availability in the preparation room and treatment serve as proxies. The measures and the specific guidance informing the measure (as relevant) are summarized in Table 1 and visually depicted in Fig. 1. We added a prompt about glove use, which is not a substitute for handwashing in healthcare settings(37), though there is no specific guideline for glove use. The intention was to understand the practice in these contexts, particularly in relation to handwashing.” | |
| E2b.  b. Please additionally present findings by country and by facility in the results, as this could inform more contextually-targeted recommendations. | E2  b. We report the specific responses/observations related to feeding assessment questions by Facility in supplementary table 1. We have chosen to place this in the supplement because the table is a bit cumbersome when disaggregated by facility. We created Figure 1 as a means of more clearly reporting information by facility. The traffic light format enables the optimal and suboptimal observations to emerge. And, it allows for assessment by observation (to understand the number of potentially risk behaviors per observation) as well as by facility.  In the methods, we noted that facilities were randomly labeled A-L to maintain confidentiality. We recognize that sorting by study site would enable more contextually-targeted recommendation, as is recommended. As such, we have re-formatted Figure 2 to sort the de-identified facilities by study site. While there are 3 countries, we have elected to sort India by the two states, resulting in four study sites. We have elected to keep the study sites de-identified for confidentiality. | |
| E2  c. Finally, please discuss environmental hygiene in the context of resource limitations in LMICs, highlight the need for formal guidance and policy around this topic, and provide specific recommendations for generating further evidence in this area. | E2.  c. We have made adjustments to the manuscript along these topics as advised by reviewer 3. Point-by-point responses to these and all the other comments by reviewer 3 are below. | |
| E3.  Please include the following items when submitting your revised manuscript:  a. A rebuttal letter that responds to each point raised by the editor and reviewer(s). You should upload this letter as a separate file labeled 'Response to Reviewers'.  b. A marked-up copy of your manuscript that highlights changes made to the original version. You should upload this as a separate file labeled 'Revised Manuscript with Track Changes'.  c. An unmarked version of your revised paper without tracked changes. You should upload this as a separate file labeled 'Manuscript'. | E3.  a-c. We confirm that we have included the recommended documents. | |
| **Journal Requirements** | | |
| **Comments / requested revisions** | **Authors’ response** | **Additions /edits to manuscript (as applicable)** |
| J1.  Please review your reference list to ensure that it is complete and correct. If you have cited papers that have been retracted, please include the rationale for doing so in the manuscript text, or remove these references and replace them with relevant current references. Any changes to the reference list should be mentioned in the rebuttal letter that accompanies your revised manuscript. If you need to cite a retracted article, indicate the article’s retracted status in the References list and also include a citation and full reference for the retraction notice. | J1.  We have reviewed the list and it is complete and correct.  As a note, the following citations have been added since initial submission:  World Health Organization. (2018). *Infection prevention and control assessment framework at the facility level* (No. WHO/HIS/SDS/2018.9). World Health Organization.  Vesel, L., Bellad, R. M., Manji, K., Saidi, F., Velasquez, E., Sudfeld, C. R., Miller, K., Bakari, M., Lugangira, K., & Kisenge, R. (2023). Feeding practices and growth patterns of moderately low birthweight infants in resource-limited settings: results from a multisite, longitudinal observational study. BMJ open, 13(2), e067316.  Arriola, K. R. J., Ellis, A., Webb-Girard, A., Ogutu, E. A., McClintic, E., Caruso, B., & Freeman, M. C. (2020). Designing integrated interventions to improve nutrition and WASH behaviors in Kenya. Pilot and feasibility studies, 6(1), 1-16.  Freeman, M. C., Ellis, A. S., Ogutu, E. A., Caruso, B. A., Linabarger, M., Micek, K., Muga, R., Girard, A. W., Wodnik, B. K., & Arriola, K. J. (2020). Impact of a demand-side integrated WASH and nutrition community-based care group intervention on behavioural change: a randomised controlled trial in western Kenya. BMJ Global Health, 5(11), e002806. | |
| J2.  Please provide additional details regarding ethical approval in the body of your manuscript. In the Methods section, please ensure that you have specified the name of the IRB/ethics committee(s) that approved your study. | J2.  As requested, we have provided additional details, including the names and numbers. See Ethics section. The following was added in the methods:  “(1) India Health Ministry’s Screening Committee with Indian Council of Medical Research acting as its secretariat (2019–2674); (2) Directorate of Health and Family Welfare Services, Government of Karnataka, which also covers investigators at Women and Children Hospital, Davangere and Chigateri General District Hospital, Davangere (NHM/SPM/04/2019–20); (3) Institutional Ethics Committee of KLE Academy of Higher Education and Research which also covers investigators at JN Medical College, Belagavi and KLES Dr Prabhakar Kore Hospital & Medical Research Center, Belagavi (KAHER/IEC/2019–20/D-2760); (4) Institutional Ethics Review Board of SS Institute of Medical Sciences and Research Centre (IERB/200/2019); (5) Institutional Ethics Committee of JJM Medical College (JJMMC/IEC-01/2019), which also covers investigators at Bapuji Child Health Institute and Research Centre, Davangere, Women and Children Hospital, Davangere and Chigateri General District Hospital, Davangere; (6) Research and Ethics Committee, Directorate of Health Services, Odisha state, which also covers investigators at City Hospital Oriya Bazar, Cuttack (155/PMU/187/17); (7) Institutional Ethical Committee, Sriram Chandra Bhanja Medical College, Cuttack (7188); (8) Malawi National Health Sciences Research Committee (NHSRC2019/Protocol19/03/2250-UNCPM 21905); (9) Tanzania National Institute of Medical Research (NIMR/HQ/R.8a/Vol.IX/3126); (10) Muhimbili University of Health and Allied Sciences (DA.282/298/01.C/); and (11) Harvard T.H Chan School of Public Health (IRB10-0282) which also covers investigators at Boston Children’s Hospital, Brigham and Women’s Hospital, Emory University, PATH and University of North Carolina.” | |
| J3.  Please include a complete copy of PLOS’ questionnaire on inclusivity in global research in your revised manuscript. Our policy for research in this area aims to improve transparency in the reporting of research performed outside of researchers’ own country or community. The policy applies to researchers who have travelled to a different country to conduct research, research with Indigenous populations or their lands, and research on cultural artefacts. The questionnaire can also be requested at the journal’s discretion for any other submissions, even if these conditions are not met.  Please find more information on the policy and a link to download a blank copy of the questionnaire here: https://journals.plos.org/globalpublichealth/s/best-practices-in-research-reporting. Please upload a completed version of your questionnaire as Supporting Information when you resubmit your manuscript. | J3.  As requested, we have completed and submitted the questionnaire. | |
| J4.  Please amend your detailed Financial Disclosure statement. This is published with the article. It must therefore be completed in full sentences and contain the exact wording you wish to be published.  a. State the initials, alongside each funding source, of each author to receive each grant.  b. State what role the funders took in the study. If the funders had no role in your study, please state: “The funders had no role in study design, data collection and analysis, decision to publish, or preparation of the manuscript.”  c. If any authors received a salary from any of your funders, please state which authors and which funders.  If you did not receive any funding for this study, please simply state: “The authors received no specific funding for this work.” | J4.  a-c.  The Financial Disclosure statement has been funded as follows:  a. This publication is based on research funded by the Bill & Melinda Gates Foundation  [OPP1192260] (KEAS).  b. Under the grant conditions of the Foundation, a Creative Commons Attribution 4.0 Generic License will be assigned to the manuscript. The findings and conclusions contained within are those of the authors and do not necessarily reflect positions or policies of the Bill & Melinda Gates Foundation. The funders had no role in study design, data collection and analysis, decision to publish, or preparation of the manuscript.  c. The following authors received salary support from the Bill & Melinda Gates Foundation:  Bethany A. Caruso  Uriel Paniagua  Irving Hoffman  Karim Manji  Friday Saidi  Christopher R. Sudfeld  Sunil S Vernekar  Mohamed Bakari  Christopher P. Duggan  George C. Kibogoyo  Rodrick Kisenge  Sarah Somji  Eddah Kafansiyanji  Tisungane Mvalo  Naomie Nyirenda  Melda Phiri  Roopa Bellad  Sangappa Dhaded  Chaya K A  Bhavana Koppad  Shilpa Nabapure  Saumya Nanda  Bipsa Singh  S Yogeshkumar  Katelyn Fleming  Danielle E Tuller  Katherine EA Semrau  Linda Vesel  Melissa F Young | |
| **Reviewer 1** | | |
| **Reviewer comments and requested revisions** | **Authors response** | **Additions /edits to manuscript (as applicable)** |
| R1.1.  Overall, this is an interesting article addressing a critical information gap. My primary questions / critiques relate to the methods and presentation of the results. | R1.1.  We are grateful for the reviewer’s time reviewing the paper and offering feedback for strengthening. | |
| R1.2.  Introduction: The introduction is good, but covers a wide range of topics and feels a bit unwieldy at times. Consider consolidating / restructuring the first two paragraphs to focus in on the issues presented in the manuscript? | R1.2.  We have made adjustments to the first few paragraphs as recommended. Notably, we start with focus on facilities instead of low birthweight infants to align better with the manuscript focus. | |
| R1.3.  Data collection And Analysis:  a. What informed the facility needs assessment, beyond the JMP standard questions? There are various tools available - such as the WHO IPC Readiness Assessment - that could guide this data collection. If an existing tool was used or adapted, it would be useful to include that in the methods section. Further, only the classification of WASH services is presented - what was done with the rest of the data?  b. Related: WASH data is only reported for water and sanitation. What about hygiene? If data on hygiene facilities were not collected, consider referring replacing “WASH” with “water and sanitation”. | R1.3.  a. In the intro section we, point out that there are tools to assess and guide improvements in WASH and IPC in HCFs (with citations to example), though note that there are no tools specifically for assessing IPC related to behaviors for infant feeding, and thus these are needed. The noted WHO IPC Readiness Assessment tool was not one we referenced, but one we now cite. Like the other tools we found, the WHO tool is a valuable facility level tool, but does not focus on specific assessment of infant feeding behaviors.  In the methods section we already describe the items in our tool and how they were informed. Specifically, we note that some questions for our tool come directly from a WHO/UNICEF tool as follows:  “Questions used to assess facility-level water and sanitation are among the ‘core questions’ used for global monitoring of water and sanitation in healthcare facilities by the WHO/UNICEF Joint Monitoring Programme for Water Supply, Sanitation and Hygiene for global monitoring.”  In the methods, we already describe the observation tool and how, because no similar tool already existed, it was adapted based on guidelines for infection prevention and control related to breast pump milk collection kits:  “There is no standardized tool for assessing infant feed preparation in healthcare facilities. As such, we created feeding preparation observation prompts based on relevant guidance from the Joint Working Group of the Healthcare Infection Society & Infection Prevention Society on the decontamination of breast pump milk collection kits and related items at home and in hospital.(36) Given that the guidance was created for and informed by practitioners in the United Kingdom, observation prompts were added or adapted to fit the context and research scope. For example, Guideline 1.2 emphasizes the importance of ‘good microbiological quality water’ where mothers express milk and for rinsing of feeding-related implements. Microbial water quality assessment was not possible, thus questions about water availability in the preparation room and treatment serve as proxies. The measures and the specific guidance informing the measure (as relevant) are summarized in Table 1 and visually depicted in Fig. 1. We added a prompt about glove use, which is not a substitute for handwashing in healthcare settings(37), though there is no specific guideline for glove use. The intention was to understand the practice in these contexts, particularly in relation to handwashing.”  We have now added citation the WHO IPC Readiness Assessment tool in the introduction based on reviewer feedback.  b. We appreciate the observation related to the term ‘WASH’. For the Facility-level assessments in the results, we have shifted the section heading and the table title to say ‘water and sanitation’.  We clearly address hand and feeding implement hygiene as part of the observation. That said, this comment also made us aware of a gap in our adapted observation tool. While we ask if washing hands and cleaning feeding implements with soap, we do not ask if there is soap in the room where these behaviors occur. While it may be a given in higher resourced settings that soap or other cleaning agents may be available, soap is not a given in all contexts. And, whether or not soap is available can determine if behaviors are performed optimally. We have now added a comment in the limitation section as follows:  ‘We already recognize changes needed in the tool we created. Specifically, while we ask if hand washing and feeding implement cleaning is carried out with soap, we did record if soap or cleaning agents were even available where the behaviors were taking place. Availability of soap may be expected in high resource settings, though is not guaranteed, particularly in low resource settings. Future work should record the availability of hand and implement cleaning agents given that their absence prevents optimal behaviors and requires critical facility-level action.’ | |
| R1.4.  P8, line 160: Glove use and the added prompt. It’s unclear from the text if feed preparers were or were not supposed to use gloves? Gloves aren’t mentioned in the conceptual model and the text is ambiguous about how this would be incorporated? Could this be combined with the hand washing to be a “YELLOW” if not combined with HWWS (and building on traffic light system)? | R1.4  We have aimed to provide more clarity in the text about glove use. In short, there is no guidance on glove use. There is guidance on handwashing, and glove use is not a substitute for handwashing. We aimed to clarify in the text that we were simply aiming to see if glove use was practiced in these contexts. And, if practiced, if hands were washed prior.  We appreciate the suggestion for a ‘yellow’ addition to the traffic light system, but prefer to stick to just green and red for clarity of what is optimal and what is not. We do note glove use in the traffic light figure, though, because there is no guideline indicating gloves should be used, simply record if gloves are used with a ‘yes’ and a ‘no’ and not a color. This allows the viewer to look at the responses for glove use and see also if hands were washed.  Note that we specifically report the following at the end of the results section: ‘While gloves were reported to be in use for almost half of the assessments (11/27; 41%), handwashing is still required when using gloves and did not occur in one instance when gloves use was observed. In two observations from the same facility (A), hands were neither washed nor gloved.’  The text was revised as follows:  ‘Though there is no specific guideline for glove use, we added a prompt to record glove use. Importantly, glove use is not a substitute for handwashing in healthcare settings(37), though it may be perceived as a substitute. Thus, our intention was to understand if glove use was practiced and, if so, handwashing was also carried out.’; | |
| R1.5.  Conceptual model: Unclear why both treatment and improved water source are included as two separate variable - if water is safe why is additional treatment required. I can make assumptions here, but I think it would be beneficial to make the logic here explicit. | R1.5.  We appreciate the need for clarification.  As we note in the introduction, water from an improved source, because of its design or construction, has the ‘potential to deliver safe water.’ Water from an improved source is not guaranteed to be safe. We clarified the text based on this useful comment. The following was added to the paragraph in the methods describing the In-Facility Observational Cohort for clarity:  ‘Treatment was assessed because having an improved water source on premises does not guarantee water is safe, only that its construction and design has the ability to deliver safe water.’ | |
| R1.6.  P8: Given the instructions to observe 5 feed preparations and that only 2 facilities reached this number and three facilities recorded no feed preparations - a bit more information is needed to understand the sampling here. How long were data collectors in each facility? Were feeding observations done simultaneously with other data collection? How many data collectors per facility?  Where were data collection staff located etc? | R1.8  We appreciate the need for more information about the methods.  Research teams at each facility were comprised of nurses who were trained on all the facility-based data collection tasks for the broader LIFE cohort study. They were present at facilities for the duration of the cohort enrollment (13 September 2019 and 27 January 2021). The specific number of trained nurses varied by facility and over the course of the study; facilities had from 6-12 nurses trained on facility-based research activities per site.  Some data were collected at the same time as other data collection activities. Specifically, the facility-level observations (e.g. those assessing water, sanitation, etc.) were completed as a part of a broader facility assessment that sought to understand the availability of a broader set of resources at the engaged facilities to enable their care for LBW infants (e.g., anthropometric measuring equipment, milk expression tools, medications).  Observations of infant feeding preparation were not carried out simultaneous to other data collection. These feeding preparation observations were only carried out if the there was an opportunity to observe a feed preparation AND the research team was not engaged in other research activities that were of greater priority to the parent cohort study (e.g., enrolling infants in the cohort or observing care of enrolled infants while still in the facility).    The following has been added to the text at various points in the paper for clarity (underlined is new text):  In methods:  ‘Trained data collectors used standardized tools across all facilities to record the structural, human resource, equipment, and service inputs present for mothers and their newborns at each facility. Data was collected via direct observation and in consultation with staff for confirmation. This paper specifically reports the WASH-related resources and policies documented in the facility assessments; a forthcoming paper documents the availability of other resources in the facilities (e.g., medications, milk expression tools, anthropometric measuring equipment) that support care for LBW infants. Questions used to assess facility-level water and sanitation are among the ‘core questions’ used for global monitoring of water and sanitation in healthcare facilities by the WHO/UNICEF Joint Monitoring Programme for Water Supply, Sanitation and Hygiene for global monitoring.’  ‘To fill out the tool, data collectors observed a facility staff member preparing a feed for an infant who was not receiving milk directly from the breast. The feed could be for any baby in the facility, not only those enrolled in the study cohort. Follow-up questions were asked in instances of uncertainty (e.g., if and how water is treated). Observations were carried out by members of the facility-based data collection teams, which were comprised of approximately 6-12 trained nurses ata collectors in each facility, with variability by site and over time. The facility-based data collection teams were at the facilities for the duration of the enrollment period (13 September 2019 and 27 January 2021) to engage in cohort-related research activities, including enrolling LBW infants in the cohort or carrying out observations of care received by enrolled infants at the facility. For the feed preparation observations, data collectors were advised to observe and collect data on five feeding preparations per site, when possible, ideally on different days for variability. They were instructed to carry out observations only if not engaged in other cohort research activities for which they were also responsible (e.g., cohort enrollment, other infant-care observations). They did not record who the prepared feed was intended for and, as a result, data are not linked to a specific infant. The aim was to simply gain insights about facility practices and procedures when preparing an infant feed.’  In the limitations:  ‘We were unable to complete our target number of observations across all twelve facilities as planned because data collection teams were engaged in other research activities in those locations that were prioritized. However, though 27 observations were completed across nine facilities in three countries, providing data on 270 total practices. | |
| R1.7.  Page 9, line 189: Facilities were labeled A - L (not F). Consider breaking at line 184 for a new paragraph and starting this new paragraph with “for all data..” Or something to indicate that this was the approach used for all data collected, not just feeding observations. | R1.7  We have re-labled the facility IDs and have also started a new paragraph as suggested for clarity. | |
| R1.8. Why were no feeding preparations observed in facilities J - L? | R1.8  We clarify in our response to point 6 above that observations were not able to be completed in all locations because the data collection teams were engaged in other research activities while in the facilities that were prioritized (e.g., enrolling infants in the cohort). Please see responses to query R1.6 above, including how we address this point in both the methods and the limitaitons sections. | |
| R1.9.  P2, last paragraph: Authors report a total of 27 observations completed. Page 4 - the denominator is reported as 28. Not sure where the extra observation came in? | R1.9  This was a typo and we appreciate the catch. We have re-checked and changed the numerator and denominator as appropriate. The percent remains the same. The text now reads with the numbers as follows:  ‘Specifically, an optimal water treatment practice was reported across all facilities (9/9) and across almost all observations (26/27; 96%) (1 observation with missing data), despite a suboptimal preparation room water source (water that is stored in the room from an outside source) being reported across three facilities. Similarly, across almost all observations (25/27; 93%), feeding supplies were reported to be cleaned after each use.’ | |
| R1.10  P3: suggest moving Table 2 to earlier (maybe just here for page break reasons, but it was a bit confusing to suddenly jump back to previously read data) | R1.10  We did place the table where it was for page break reasons as the reviewer notes. We did move it forward (not in track changes) to aid the reader. We expect that, if accepted, the editorial office will move further. | |
| R1.11  P5, first paragraph: this paragraph uses the word reported. Were these practices not observed? | R1.8  We have now clarified in the methods that the facility-level data collection involved both observation and consultation with facility staff for confirmation. For example, observation of water facilities is possible, though data collectors gained information from staff regarding the presence of policies or typical water treatment practices. We have clarified the results to have the word’ reported’ only as applicable.  The addition to the methods is as follows:  ‘Data was collected via direct observation and in consultation with staff for confirmation.’ | |
| R1.12  There’s quite a bit of missing data - table 2 combines “missing data” with “not observed” but these are two very different things. Would be useful to differentiate between the two when possible. | R1.12  Our table 2 has no missing data and no ‘not observed’ data.  We assume the reviewer may mean the ‘traffic light’ visual (Figure 2). All missings in a direct observation are in fact ‘Not observed’ so we have adjusted the language for clarity based on the recommendation.  We have also color-coded those facilities where direct observations of feeding preparation did not occur at all for further clarity. | |
| R1.13  Discussion: In the end - what’s the contribution of the sanitation data? It’s only in the tables and its importance is not reflected back in the discussion. Considering all of the facilities had both improved sanitation facilities and improved water sources - what is the data contributing to the manuscript? | R1.13  The discussion section does include mention of the sanitation data, though we have added a sentence for further clarity. The primary point is that water and sanitation are assessed at the facility level as potential proxies for a facility’s resources and ability to prevent and control infection. All facilities in our study had improved water and sanitation access, though facility level access did not ensure optimal behaviors. The following is from the discussion (with the new contribution underlined):  ‘All 12 facilities assessed in this study had improved water and sanitation facilities, which are necessary for infection prevention and control, but not sufficient, particularly if services are not accessible where needed (22, 39). As Burki (2019) notes, access to basic WASH, though critical, does ‘not necessarily equate to safe services.’ Our research supports Burki’s point, having identified various types of unsafe practices related to newborn feeding within the facilities despite facility-level WASH access. In other words, assessing water and sanitation access alone as proxies for assessing facility-level resources to prevent and control infection is insufficient and potentially misleading. For example, this work further demonstrates that it is critical for a water source to not only be available at the facilities, but where hygiene behaviors are to occur.’ | |
| **Reviewer #2** | | |
| **Reviewer comment** | **Authors response** | **Added text (as applicable)** |
| R2.1  Congratulations to the team, the information adds value to change of practice and behaviours towards safe infant feeding in our health facilities. Authors are contributing to an important piece of baseline information alerting more actions needed | R2.1  We are very grateful to the reviewer for the feedback provided and the support for this work. | |
| R2.2  Study setting Line 122 – 124 states that, The LIFE study was conducted in 12 secondary and tertiary level facilities (both public and private) in four urban sites across three countries: (1) Karnataka and (2) Odisha states in India;(3) Lilongwe, Malawi; and (4) Dar es Salaam  a. The 4 urban sites are confusing, I suggest to just mention 3 countries, I presume that all 12 facilities were in urban settings.  b. Add rationale of including public and private sites | R2.2  a. We appreciate that the current reader presumed that the sites were urban, but not all readers may have the same assumptions. It is important for us to make it clear that all sites were urban, so we will keep that distinction. We have discussed the sites similarly across other papers (noted below), thus also want to maintain our description for consistency.  Examples of other papers where study sites described:  Vesel, L., Spigel, L., Behera, J. N., Bellad, R. M., Das, L., Dhaded, S., ... & Semrau, K. E. (2021). Protocol: Mixed-methods, descriptive and observational cohort study examining feeding and growth patterns among low birthweight infants in India, Malawi and Tanzania: the LIFE study protocol. BMJ Open, 11(12).  Vesel, L., Bellad, R. M., Manji, K., Saidi, F., Velasquez, E., Sudfeld, C. R., ... & Semrau, K. E. (2023). Feeding practices and growth patterns of moderately low birthweight infants in resource-limited settings: results from a multisite, longitudinal observational study. BMJ open, 13(2), e067316.  b. We had specific inclusion or exclusion criteria for public or private institution. We note specifically In the study setting section of the methods that selection was based on delivery volume, capacity to care for LBW infants, and facility leadership willingness to engage in the study. Of those that fit these criteria, the final mix included both private and public facilities. We have changed where we mention this to clarify to readers that being a public or private site was not factored into the selection criteria. The below indicates how we discuss selection in the methods, with underlined text being new:  ‘Facilities were selected based on their delivery volume, capacity to care for LBW infants in the first days of life, and the willingness of facility leadership to engage in the study; both public and private facilities were included.’ | |
| R2.3  General comments a. It will be useful to analyze and show results per country and per facility when needed. Results may have been pulled to certain facilities. Observations analysis per facility per country is important – this may lead to country specific recommendations/observations rather than general.  b. Figure 2 – couldn’t be read, the font is too small and unreadable | R2.3  a. We report the specific responses/observations related to feeding assessment questions by Facility in supplementary table 1. We have chosen to place this in the supplement because the table is a bit cumbersome when disaggregated by facility.  We created Figure 2 as a means of more clearly reporting information by facility. The observations are already sorted by facility. The traffic light format enables the optimal and suboptimal observations to emerge. And, it allows for assessment by observation (to understand the number of potentially risk behaviors per observation) as well as by facility.  We appreciate the recommendation to sort by country. Given that we have two very different sites in India, we think that sorting by study site would enable more contextually-targeted recommendation, as is recommended. As such, we have re-formatted Figure 2 to sort the de-identified facilities by study site. While there are 3 countries, we have elected to sort India by the two states, resulting in four study sites. We have elected to keep the study sites de-identified for confidentiality.  b. We have made adjustments to the Figure 2 to make more legible and hope that that the revisions enable a better review and enhanced readability. | |
| R2.4  Other comments  a. Among 270 behaviours assessed across the 27 feeding preparation observations, 46 (17.0%) practices were carried out sub optimally, including preparers not handwashing prior to preparation, and cleaning, drying, and storing of feeding implements in ways that do not effectively prevent contamination à What were other behaviours observed to be suboptimal?? Only hand washing was discussed  b. Line 179 -181: ‘We calculated the frequency of the various WASH-related practices for feed preparations overall and by facility. For each observation, we also determined which practices carried out as part of the feeding preparations were optimal and which were suboptimal based on the guidelines’ àThis was not pictured well in the results (Table or Figure), may be its in Figure 2 – which is difficult to view | R2.4  a. In addition to hand washing being sub-optimal, we do discuss the other behaviors. Specifically, we note that cleaning behaviors were sub-optimal, as was drying, and storing. See the final two paragraphs in the results.  b. We visually show each behavior per observation in Figure 2 as optimal or sub-optimal. We hope the revisions to the figure have enabled clarity. Further, we note in the text that ‘specific responses/observations related to feeding assessment questions are reported in Supplemental Table 1 by facility.’ We present all data in the paper in multiple formats to enable reader review. | |
| R2.5  Limitation Hawthorn effect Line 172 – 173 reads ‘Data collectors carried out observations opportunistically as they noticed a feed being prepared for any baby in the facility’  a. What does opportunistically mean?? How was hawthorn effect controlled?  b. What was the interobserver variability?  c. Distribution of observations per country? Per facility? | R2.5  a. Opportunistically means ‘as opportunities arose’. We have taken the word out as we realize it is redundant with the explanation that follows. It now reads:  ‘Data collectors carried out observations as they noticed a feed being prepared for any baby in the facility, not only those enrolled in the study cohort.’  We have also noted the Hawthorne effect now in the limitaitons as follows:  ‘As with any data collection involving observation, Hawthorne effect (those being observed changing behavior due to awareness of being observed) is always a potential limitation. Those preparing feeds could have changed behavior, though we assume they would be more likely to perform a behavior optimally when observed. As such, if the Hawthorne effect is a factor in this study, we most likely under-observed sub-optimal behaviors, resulting in conservative estimates of the proportion that were suboptimal.‘  b. We were not able to assess interobserver variability. We would only be able to calculate this if we had the same two observers carrying out all observations across all sites and comparing their observations. Data collection was assessed across multiple sites that were geographically very far so this was not methodologically possible.  c. The number of observations by facility and by country are made clear in Figure 2 and also in supplemental table 1. | |
| **Reviewer 3** | | |
| **Reviewer comment** | **Authors response** | **Added text (as applicable)** |
| R3.1  This is a novel and useful study on infection prevention and control conditions and behaviors and the relationship and infant feeding provision in healthcare facilities across multiple study sites. I do not have any major concerns with the manuscript, and believe it sound and of value. Please see attached a few comments which might be useful (or not!) to perhaps expand thinking and tweak the manuscript slightly. My main point is I would discuss environmental hygiene and limitations of what we expect given resource availability; also to highlight further the lack of formal guidance and policy around this topic and to emphasise the need for this and how we might go about generating further evidence towards formal policy, how to achieve etc. Thank you. | R3.1  We very much appreciate the time and value feedback provided by the reviewer.  We have addressed all the comments in the attached document, which include all of the reviewers noted points in this comment. Specifically, we have expanded the discussion on what we may expect given resource availability, note the need for formal guidance and improved tools. And suggest ideas for further research and evidence needed.  Please find below each of the comments from the PDF and our specific responses. | |
| R3.2  Intro: lines 64-66: Is it worth further clarifying the cyclical relationship between early-life infection and growth? This might already be obvious | R3.2  We have added the following (new text underlined):  ‘The vulnerabilities faced by LBW infants underscore their need to receive care in environments that not only support their growth and development, but also limit their exposure to pathogens, which may be particularly harmful to LBW infants who have less well developed immune systems making them more susceptible to infection and impacts on future growth and development.’ | |
| R3.3  Line 72. Re: ‘Readiness’- in terms of? | R3.3  Here we have added ‘(e.g., resources)’ next to the word ‘readiness’. | |
| R3.4.  Line 73-53:  Might be a useful summary point at the end of this section (just a thought) | R3.4  We think we have captured this well here and in the lines that follow. | |
| R3.5  Line 88-89:  Do you mean they are able to, but are not? Or they cannot? | R3.5  The reviewer has highlighted the following line that describes improved water sources: ‘which by design or construction have the potential to deliver safe water.’ This is cited from the WHO/UNICEF Joint Monitoring Programme as part of the definition for an improved water source. I have now added quotes to the specific terms used. It means what it says. It has the potential to deliver it, but is not guaranteed. | |
| R3.6  Line 91-92:  Yes - but we can also expect these behaviours to take place when the governing environment does not/cannot support it. This is unfair and pushes responsibility onto the HCP/parent/community member for a large burden of infection that is impossible to avoid with an over-reliance on personal-level measures | R3.6  I think that the reviewer is actually making a similar argument as we make in the paper. Specifically, we note that even having water does not mean that people will use it to wash their hands. We do not disagree that personal-level measures are less ideal that making structural and environmental changes. However, while it is possible to remove the need for individuals to treat water (behavior) if safe, piped water is provided directly to the home, there is no environmental or system level intervention that can eliminate the need for handwashing. People simply need to do it. Our study takes place in specialized health facilities, so we do not think it inappropriate or ;unfair’ as the reviewer write, to suggest that handwashing is their responsibility. | |
| R3.7  Line 102-103:  If the environment enables it | R3.7  The specific comment provided by the reviewer does not seem complete, so we have not made any changes. If the reviewer is referring to environmental conditions, we do assess those so do not think any further change is warranted here. | |
| R3.8  Methods:  Are you able to give slightly more background info on cohort numbers, dates, timelines etc. in some section here? | R3.8  We deliberately do not go into great detail about the cohort study as we do not want to confuse or distract the reader. That said, we have added text to guide the readers to where more information can be found based on the reviewer comment. Text in the paper is as follows, with new text underlined:  ‘This study was a component of the Low Birthweight Infant Feeding Exploration (LIFE) study, an observational cohort study that aimed to document the current feeding practices and growth patterns among LBW infants in LMICs in order to inform feeding interventions. Additional information about the cohort study methods can be found in the protocol(34) and in the paper documenting infants’ feeding practices, growth patterns, and risk factors for growth outcomes at six months(35). This paper reports findings related to WASH and IPC from two data streams: the facility needs assessment and the in-facility observational cohort.’ | |
| R3.9  Study Setting  Line 122:  might it help to give examples of what these are? | R3.9  We have made the following edit (new text underlined):  ‘The LIFE study was conducted in 12 secondary and tertiary level facilities (i.e., facilities where patients are referred by their primary care provider for more specialized service) in four urban sites across three countries…’ | |
| R3.10  Line 132:  Might it be beneficial to separate this out? I am slightly slow to follow exactly what was recorded (might be me!) but e.g., below seems to merge policy with resource | R3.10  We document the availability of both policies and resources *at the facility level.* These are simply noted in this section as they are collected as part of the facility-level assessment. We simply note an absence or presence of policies and various resources as part of our tool. These are separated out and discussed independently in the results. This would have become more clear to the reviewer in the sections that follow. | |
| R3.11  Line 138-140:  as above | R3.11  As above. | |
| R3.12  Line 151:  this is an important point which underlies a lot of the need for this research and might benefit from emphasizing it | R3.12  We agree that this is important and also focus much of the discussion on the need for standardized guidance. Further, we note also in the discussion that the tool we adapted was useful, but even after initial use can be improved. We offer suggestions for improvements in the limitations section. Below is text from the discussion with new text underlined:  ‘We created a novel, guidance-informed tool to assess infection risk related to infant feeding that allowed us to present data by observation, allowing identification of trends by and across facilities and study sites. The guidance informing the tool, though informative, was created for high-income settings and was specific to decontamination of breast pump parts in healthcare settings. Given that feeding and feed preparation is a complex, multi-step process(29), further work should be done to improve both the tool and conceptual model to ensure that they both comprehensively capture all conditions and behaviors relevant to infant feeding in healthcare settings. We already recognize changes needed in the tool we created. Specifically, while we ask if hand washing and feeding implement cleaning is carried out with soap, we did record if soap or cleaning agents were even available where the behaviors were taking place. Availability of soap may be expected in high resource settings, though is not guaranteed, particularly in low resource settings. Future work should record the availability of hand and implement cleaning agents given that their absence prevents optimal behaviors and requires critical facility-level action. Unstructured observations across multiple settings could be carried out as a first step to inform further modifications to the tool.’ | |
| R3.13  Line: 155-156  This is important as mentioned above in comments that we perhaps should not try to impose an impossible standard | R3.13  We agree that it is important to not impose a possible standard and have devoted parts of the discussion noting that overall guidance is needed that is also appropriate for contexts and resources available. | |
| R3.14  Line: 161-163  Is this standard use? Not for all settings I think and depends on need - over-use of gloves can lead to cross-contamination (we saw this particularly during covid)  https://www.england.nhs.uk/atlas_case_study/the-gloves-are-off-campaign/ | R3.14  We have updated the methods here for clarity, also noted by another reviewer. There is no guidance on glove se. However, we observed glove use just to understand if the practice was carried out, and we highlight that glove use is NOT a substitute for handwashing. The update is as follows (new text underlined):  ‘Though there is no specific guideline for glove use, we added a prompt to record glove use. Importantly, glove use is not a substitute for handwashing in healthcare settings(37), though it may be perceived as a substitute. Thus, our intention was to understand if glove use was practiced and, if so, handwashing was also carried out.’ | |
| R3.15  Line: 182-183  Probably best, but does lead to a slightly negative skew of the view of things - and might this downplay where positives do occur and what can be built on? | R3.15  We understand the reviewer’s point, but the disagree that this is a skew of the findings because the suboptimal behavior could be enough to lead to pathogen exposure and then infection. Certainly, if someone in a hospital gave a patient a perfectly clean glass, but then also put water in that glass that was contaminated with cholera, it would be appropriate to focus energy on the water quality as that is where the risk lies. Further, all | |
| R3.16  Line 277-279  Expanding on my point above, it seems that even in the presence of proper resources and a good governing hygienic environment, infection can be introduced with poor practices and a lack of policy/guidance. That's a salient point, and I think it could be emphasised to a greater degree. I am totally onboard with the premise that we need greater governance and accountability to improve outcomes where there is sufficient infrastructure  However I'd also ask but doesn't that occur around the world too in upper-income contexts? What level should we be expecting? Maybe not totally relevant or interesting but just a thought | R3.16  We certainly agree with the reviewer’s point. And also agree that there may be similar issues in well-resourced settings. Indeed, nurses and doctors in well-resourced hospitals certainly do not wash their hands 100% of the time.  The comment highlights a line in the first sentence of the introduction paragraph with a flag to expand the point. In the intro to the discussion we simply lay out the key points, and we do expand on this point considerably in the next paragraph that follows. We have pasted that paragraph below, and have also underlined new additions to the text that further the point:  ‘All 12 facilities assessed in this study had improved water and sanitation facilities, which are necessary for infection prevention and control, but not sufficient, particularly if services are not functional, accessible, designed appropriately, or located where needed(22, 39). As Burki (2019) notes, access to basic WASH, though critical, does ‘not necessarily equate to safe services.’ Our research supports Burki’s point, having identified various types of unsafe practices related to newborn feeding within the facilities despite facility-level WASH access. In other words, assessing water and sanitation access alone as proxies for assessing facility-level resources to prevent and control infection is insufficient and potentially misleading. For example, this work further demonstrates that it is critical for a water source to not only be available at the facilities, but where hygiene behaviors are to occur. We found that handwashing did not occur before feeding preparation in 3/27 (11%) observations, all clustered in just two healthcare facilities. In three instances, we observed that water was not available in the room where the feed was being prepared, potentially serving as the primary barrier for handwashing in those instances. Having the necessary resources—including water, soap, and disposable towels for drying—within reach in food preparation areas creates the ideal environment for handwashing prior to handling food and implements(29). Of course, not all facilities will have the resources to make water, soap, and disposable towels in each room, but recommendations should still be made clear, with options for less resourced areas (e.g., air drying is also a safe drying strategy; cloth towels should be avoided). For the other observations where handwashing did not occur, water was observed to be available in the room, clearly showing that resource availability alone is not enough to ensure optimal behaviors. Additionally, given that we observed the use of gloves, without handwashing prior, the availability of resources may hamper proper hygiene. Gloves are not a replacement for hand hygiene as they can break and expose contaminated hands(40).’ | |
| R3.17  Line: 286  or always functional, or delivering as intended | R3.17  Point noted. The line reads as follows (new text underlined):  ‘All 12 facilities assessed in this study had improved water and sanitation facilities, which are necessary for infection prevention and control, but not sufficient, particularly if services are not functional, accessible, designed appropriately, or located where needed.’ | |
| R3.18  Line: 359  Do you think there's any responsibility for care beyond the healthcare facility? Just a thought | R3.18  We absolutely agree that there is responsibility for care beyond the health facility. We have added the following in the discussion:  ‘Finally, improvements to feeding behaviors are critical in healthcare settings, though no less important when infants are at home. This research focused on behaviors in healthcare settings because infants are increasingly delivered in a facility where they may be at risk of HCAIs and may be in particular need of feeding assistance if LBW. Those working in facilities could be models for mothers and other caregivers. As such, they have an opportunity to model and teach optimal behaviors so they may be taken up beyond the healthcare setting. Further work, however, is needed to encourage optimal feeding strategies for infants in the home that are both nutritious and safe, and interventions like those in Kenya focusing on ‘mealtime’ safety may be a useful guide.’ | |
| R3.19  Line 362-363:  I always wonder about the question of adapting according to context. Does that mean we level up or down (or neither?) Obviously we have to work within the limitations and resource availability of the context, but I wonder if there's something more about homogenizing levels of service / policy / guidance | R3.19  The statement itself is written as neutral—neither leveling up nor leveling down—and that is how it is intended. We simply mean that that some basic guidance about what should be done (e.g. handwashing pre-feeding) can be universal, but the specific means of operationalizing the guidance in each context can vary based resources available and identified barriers and motivators to handwashing in a particular setting. We have updated the text as follows (underlined text is new):  ‘Both resources and context-specific behavioral guidance and behavior change strategies are needed to ensure optimal hygiene behaviors related to child feeding to prevent pathogen introduction and infection, as has also been observed in household contexts(41). For example, general guidance should be clear about the need to wash hands before handling feeding implements, but may be unfeasible if overly prescriptive by requiring a water source with a tap in each health facility room. Context-specific behavior change programming to improve handwashing should be informed by resources available as well as the identified barriers and motivators to performing hand hygiene in a specific setting.’ | |
| R3.20  Line 378:  You state that:  'Facilities were selected based on their delivery volume, capacity to care for LBW infants in the first days of life, and the willingness of facility leadership to engage in the study'  - therefore might there be a bias in the selection of sites in that they were already possibly fairly well-equipped to start, and if facility leaders were happy for the study to take place it might be because they knew that? | R3.20  This is an important point. The limitation section was expanded. The text now reads as follows (underlined text is new):  ‘Findings may not be generalizable to other locations as the facilities engaged are in urban settings, and there was also the potential for observer bias. Further, by nature of their eligibility for inclusion in the broader cohort study, the facilities themselves had the capacity to care for LBW infants in the first days of life and were willing to engage in the study, thus may have been fairly well-equipped compared to other facilities in the area.’ | |
| R3.21  Line 387:  Might there have been the potential for the hathwthorne effect? or do you think HCPs wouldn't have modified their behaviour as they wouldn't have been able to have done anything differently anyway? (this might be what you mean by observer bias, so sorry if asking you to repeat but expansion may be merited) | R3.21  The point about Hawthorne effect is important, and also raised by another reviewer. We have added the following to the limitations section:  ‘As with any data collection involving observation, Hawthorne effect (those being observed changing behavior due to awareness of being observed) is always a potential limitation. Those preparing feeds could have changed behavior, though we assume they would be more likely to perform a behavior optimally when observed. As such, if the Hawthorne effect is a factor in this study, we most likely under-observed sub-optimal behaviors, resulting in conservative estimates of the proportion that were suboptimal.’ | |
| R3.22  Figure 1:  Not best quality but readable | R3.22 and R.23  We have updated both images and hope they are more readable now. | |
| R3.23  Figure 2  Not possible to view - poor image quality |  |  |
